# Evaluating the Effectiveness of an Educational Awareness Program Through Knowledge Change: A Cross-Sectional Study Promoting Breast Awareness

**DOI:** 10.7759/cureus.89378

**Published:** 2025-08-04

**Authors:** Miyuki Yamamoto

**Affiliations:** 1 Department of Nursing Science, Hirosaki University Graduate School of Health Sciences, Hirosaki, JPN

**Keywords:** awareness education, breast awareness, breast cancer subtypes, hereditary breast and ovarian cancer, sustainability

## Abstract

Objective

In 2020, breast cancer was the most commonly diagnosed cancer among women in Japan. Its incidence begins to rise in the late twenties and reaches a first peak in the late forties. Therefore, fostering sustainable preventive health behaviors from a younger age is crucial. This study aims to evaluate the effectiveness of an educational awareness program on breast cancer from a long-term perspective by comparing knowledge levels before the intervention, immediately after, and one month following the intervention, with the goal of promoting breast awareness.

Methods and materials

This was a cross-sectional study conducted at three time points: before the breast cancer educational intervention, immediately after, and one and a half months post-intervention. A self-administered questionnaire was distributed to 82 first-year students enrolled in the Nursing Course at the School of Health Sciences, Hirosaki University, Japan. Data from 55 students (valid response rate: 64.7%) who completed all three surveys were analyzed. The number of respondents at each time point was: 67 students before the intervention (response rate: 81.7%), 72 students immediately after (response rate: 87.8%), and 63 students one and a half months later (response rate: 76.8%). Ultimately, data from the 55 students with no missing values across all three surveys were included in the analysis. The questionnaire covered topics such as breast cancer subtypes, hereditary breast and ovarian cancer (HBOC), cancer staging, peak age of incidence starting at 40, and invasive vs. non-invasive cancer.

Results

In the question “Which of the following is considered early-stage cancer?”, no significant difference was observed among students before the intervention, on the day of the intervention, or immediately after the intervention. Regarding the question “At what age range does the first peak in the incidence of breast cancer occur in women?”, scores significantly decreased immediately after the intervention and one and a half months later (p<0.001). Due to the low scores obtained, the present results suggest that a single awareness education session was not sufficient for a thorough understanding of the peak age for the development of breast cancer.

Conclusion

While the educational awareness program that provided knowledge as a preliminary step towards breast awareness showed some effectiveness immediately after the intervention, the present results indicate that a single session was insufficient for sustaining knowledge over time. Future educational efforts need to emphasize the peak incidence age of 40 years and incorporate repeated sessions in order to enhance the long-term retention of knowledge.

## Introduction

In 2020, according to Japan’s National Cancer Registry Data, breast cancer was the most frequently diagnosed cancer among women. The incidence of breast cancer begins to increase in women in their late 20s, and the “first peak” in incidence is observed in the late 40s [1]. In Japan, the number of breast cancer patients among the adolescent and young adult (AYA) generation, defined as individuals aged 15 to 39, is on the rise, with approximately 4,000 to 5,000 new cases reported annually, accounting for about 5% of all breast cancer cases [2]. Clinical data on breast cancer in the AYA population show that the disease tends to be more advanced at diagnosis and more likely to exhibit aggressive phenotypes. Although rare, AYA breast cancer is also strongly associated with hereditary germline mutations, including hereditary breast and ovarian cancer (HBOC), such as familial and hereditary forms of the disease [3]. With advances in medical science, subtypes that play a crucial role in prognosis have been revealed because they have different outcomes and varying drug sensitivities [4,5]. In Japan, breast cancer has a higher survival rate than other cancers [1].

Additionally, although rare, HBOC may result from mutations in the BRCA1 and BRCA2 genes [6]. These genes are inherited from either parent with a 50% probability, regardless of sex. While male carriers have a low risk of developing breast cancer, they may still pass the gene mutation on to their children with a 50% probability [7]. Therefore, breast cancer awareness education needs to be provided regardless of sex to enhance understanding of the biological characteristics of breast cancer. Gaining even a basic understanding of such established information may help promote preventive health behaviors beginning in the AYA generation.

When conducting awareness education, it is important to clarify the difference between “breast awareness” and “breast self-examination” for the target audience. Unlike breast self-examination, which is positioned as a screening activity, breast awareness refers to being regularly attentive to the condition of one’s own breasts and consciously incorporating that awareness into daily life [7]. This concept began spreading from the United Kingdom in the early 1990s [8]. In Japan, organized breast cancer screening is available for women aged 40 and older; however, the concept of breast awareness is considered applicable to early detection among the AYA generation and to issues related to hereditary breast cancer, which are not covered by such programs [9]. In contrast, breast self-examination is defined as a screening behavior focused on detecting lumps, examining, and diagnosing abnormalities [7]. Therefore, it is essential for individuals to receive educational programs incorporating breast awareness from the AYA stage, as this approach helps them understand that breast cancer symptoms can vary widely, not limited to the presence of lumps, and encourages appropriate preventive behavior.

Previous studies have reported numerous educational intervention programs targeting women, including those in the AYA generation. However, most of these studies focus exclusively on women and primarily emphasize breast self-examination or the promotion of positive health beliefs related to breast cancer [10-13]. According to prior research, many individuals reported that they had not had regular opportunities to receive educational programs that raise awareness or provide knowledge about breast cancer [14]. There is scientific evidence supporting the prevention of breast cancer through appropriate diet, lifestyle habits, and management of chronic conditions [15]. However, rather than conveying advanced medical knowledge, providing even basic awareness, as everyday individuals, such as understanding the existence of various breast cancer subtypes or HBOC, which differ by person regardless of gender, may help individuals recognize changes in their own breasts as part of daily life [7]. Breast awareness does not promote any special techniques or procedures but focuses on gaining knowledge about breast health [9,16,17]. Despite this, awareness of breast awareness in Japan remains low. Therefore, it is essential for individuals to receive awareness education programs from the AYA stage to promote long-term preventive health behaviors.

Since the incidence of breast cancer begins to rise in women in their twenties, the authors sought to understand the level of knowledge regarding breast health among individuals in this age group. In 2019, a survey on breast cancer knowledge and awareness-related behaviors was conducted among 421 students at Hirosaki University [18]. Subsequently, slide-based educational material (Microsoft PowerPoint) was developed, and its effectiveness was evaluated [19]. The previous survey revealed that, although many participants expressed a desire to learn more about breast cancer, opportunities to acquire such knowledge were limited [18,19].

Purpose of the study

To enable the AYA generation to practice breast awareness sustainably from a long-term perspective, it is essential to provide them with at least a minimal level of foundational knowledge. Without this, sustaining the behavior may be difficult. It is also important for individuals to understand that breast cancer presents with characteristics that vary by person, regardless of gender. Furthermore, understanding how people obtain information and the extent of their comprehension is critical when developing future educational programs aimed at promoting the widespread adoption of breast awareness.

Therefore, the aim of this study is to evaluate the effectiveness of an educational awareness program targeting first-year nursing students, individuals who are close to the general population and do not yet possess specialized medical knowledge. The findings are intended to serve as foundational data for developing educational strategies that foster sustainable health behaviors.

## Materials and methods

Study design

This was a cross-sectional study that evaluated and compared breast cancer awareness at three time points: before the educational intervention, immediately after the intervention, and one and a half months after the intervention.

Survey overview

Prior to conducting this study, we determined the required sample size. Based on a preliminary survey conducted in 2022 and findings from previous studies [20], we calculated the necessary sample size using EZR (Easy R version 2.9.5), a statistical analysis tool developed from R Commander. Using the mean and SD from prior data, we set the power (1 - β) at 0.8 and the significance level (α) at 0.05. The calculated required sample size was 73. Taking into account an anticipated response rate of 85%, the adjusted target sample size was estimated at 83. Since 82 students were enrolled in the lecture course relevant to this study, the survey was conducted with all 82 participants.

A self-administered questionnaire was distributed to 82 first-year students in the Nursing Course at the School of Health Sciences, Hirosaki University in Japan. First-year nursing students were selected as the target population because, although they have an interest in nursing and health-related topics that may make them more receptive to key concepts, they have not yet acquired specialized knowledge. This places them closer to the perspective of the general public, making them suitable for evaluating the effectiveness of the educational intervention.

The pre-intervention questionnaire survey was conducted on December 7, 2023, after the 9th and 10th lectures on “Maternal and Child Health” in the Community Health course, one week before the awareness education session. The pre-intervention questionnaire included questions on age, sex, whether the respondent knew someone who had breast cancer, and whether they performed self-examinations. This questionnaire (Appendix 1) was distributed to participants, completed, and then collected.

On December 14, 2023, the awareness education session was conducted for approximately 15 minutes within the “Adult Health” course and was immediately followed by the post-intervention questionnaire survey. The content of the awareness education program is summarized in Table 1. A total of 12 PowerPoint slides were developed for the lecture. Immediately after the educational session, a questionnaire was administered to assess participants’ understanding of the program content (Appendix 2).

**Table 1:** Overview of lecture content using slide materials.

Explanation of Breast Cancer (PowerPoint Slides; Duration: Approximately 15 Minutes)
Slide 1: Structure of the Breast
Slide 2: What Is Breast Cancer?
Slide 3: Progression of Breast Cancer
Slide 4: Classification of Breast Cancer Stages (Stage, Invasiveness, Progression)
Slide 5: Breast Cancer Subtypes
Slide 6: Types of Breast Cancer (e.g., Ductal Carcinoma In Situ, Lobular Carcinoma In Situ, Invasive Ductal Carcinoma, etc.)
Slide 7: Dense Breast Tissue
Slide 8: Genes
Slide 9: BRCA1 and BRCA2 Genes
Slide 10: How Genetic Mutations Are Inherited from Parent to Child
Slide 11: Characteristics of Hereditary Breast and Ovarian Cancer Syndrome (HBOC)
Slide 12: How to Perform a Breast Self-Examination

Another questionnaire survey was conducted on February 1, 2024, one and a half months after the intervention. The questionnaire, which included items 7 to 11 from the previous survey, was distributed, completed, and collected.

Implementation and evaluation of the program

The evaluation of the awareness education program using slide materials focused on the continuity of knowledge retention.

Five questionnaire items on comprehension were designed to evaluate the effectiveness of the lecture [15]. Specifically, the questionnaire assessed participants’ understanding of essential breast cancer knowledge required for “breast awareness,” including the following key concepts: breast cancer subtypes, cancer staging, invasive vs. non-invasive cancer, peak incidence age, and HBOC. These items reflected the core content of the educational program.

Method of evaluating the questionnaire survey: survey items and knowledge comprehension

To standardize the concepts embedded in the slide materials from the perspective of everyday individuals, five questions were developed to assess comprehension of the content delivered in the awareness education program. These questions were designed to convey essential knowledge related to breast cancer in a way that is accessible to non-experts. Regarding content validity, the materials and questions were reviewed multiple times based on prior studies, with advice obtained from medical specialists in breast cancer research. Additionally, feedback was incorporated from presentations at academic conferences, where subject-matter experts provided comments and suggestions.

Questionnaire items were designed as true-or-false questions. Each participant’s response to each question was scored as either 0 or 1. A score of 1 was considered correct and evaluated as “understood,” while a score of 0 was considered incorrect and evaluated as “not understood.” Therefore, for each item, if the average score was close to 1, it was interpreted as “well understood,” whereas if the average score was closer to 0, it was evaluated as “not well understood.”

When participants were asked what affects the division of 60-70% of breast cancer cells, a score of 1 was given for selecting estrogen, while a score of 0 was given for selecting progesterone or follicle-stimulating hormone [7]. To assess knowledge of breast cancer staging, participants were asked about the classification of breast cancer stages. A score of 1 was assigned for selecting “Stage 0 to IV,” while a score of 0 was assigned for “Stage 0 to II” or “Stage 0 to III” [1].

Regarding early-stage cancer, participants were asked to identify whether non-invasive or invasive cancer represents an early-stage diagnosis. A score of 1 was assigned for selecting non-invasive cancer, while a score of 0 was assigned for selecting invasive cancer [21]. To assess knowledge of breast cancer incidence, participants were asked to estimate the age range at which the first peak in the incidence of breast cancer occurs in women in Japan. A score of 1 was given for selecting the 40s, while a score of 0 was given for selecting any other age range [7].

Additionally, participants were questioned about HBOC and whom it affects [6,7]. A score of 1 was assigned to those selecting “Both sexes,” while a score of 0 was assigned for selecting “women only” or “men only.”

The correct answers to the questionnaire are provided in Appendix 1. Total scores were calculated by summing the responses, and the mean and standard deviation were obtained. Cronbach’s alpha, which indicates the reliability of the data, was 0.61 in this study.

Analysis method

Statistical analyses were conducted using simple aggregation, descriptive statistics, and one-way repeated measures ANOVA. The Bonferroni method was applied for multiple comparisons of within-group changes over time.

Ethical considerations

Before completing the questionnaire, participants were informed that their participation in the study was voluntary and that they had the right to refuse. It was also explained that refusal to participate would not result in any disadvantage. The purpose and objectives of the study were thoroughly explained to the participants both in writing and verbally at the beginning of the lecture, and informed consent was obtained. Consent was considered given upon the voluntary submission of the completed questionnaire. The questionnaire included only student identification numbers, which were subsequently detached and encrypted to ensure anonymity. The encrypted data were used for analysis. This study was approved by the Ethics Committee of the Graduate School of Health Sciences, Hirosaki University (Approval No. 2021-044).

## Results

A total of 55 students completed all three surveys (valid response rate: 67.1%).

Basic attributes

Among the respondents, 7 were men (12.7%) and 48 were women (87.3%). The average age of respondents was 18.7 ± 0.5 years. Seven respondents (12.7%) reported knowing someone with breast cancer, while three (5.5%) reported performing self-examinations (Table 2).

**Table 2:** Basic attributes of study participants. Age: 18.7 ± 0.5 years (mean ± SD).

Item	n	%
Sex		
Men	7	12.7
Women	48	87.3
Do you know anyone close to you who has been diagnosed with breast cancer?		
Yes	7	12.7
No	48	87.3
Have you performed breast self-examination?		
Yes	3	5.5
No	52	94.5

Information on breast cancer was most commonly obtained from television and radio, with 34 participants (61.8%) reporting these sources. The next most common sources were the internet (22 participants, 40.0%) and school classes (13 participants, 23.6%).

Only 8 participants (14.5%) had previously received breast cancer awareness education, indicating low exposure. However, 52 (94.5%) expressed a desire to attend such sessions.

In the post-awareness education survey, 50 participants (90.1%) found the difficulty level of the session to be “just right,” while 46 (83.6%) found the content “useful.” Regarding the slide materials, 46 participants (83.6%) rated them as “easy to understand,” and the handouts, which contained the same content as the slides, were also rated as “easy to understand” by the same number of participants. The flow of the awareness session was considered “just right” by 52 participants (94.5%). Regarding the ideal lecture duration, 34 participants (61.8%) preferred 30 minutes. When asked about the most common subtype of breast cancer, 25 participants (45.5%) correctly identified "luminal" as the most frequent type. In response to whether they would like to participate in another awareness session in the future, 50 participants (90.1%) answered “yes.” Furthermore, when asked if they intended to undergo breast cancer screening in the future, 53 participants (96%) responded affirmatively. Among those who indicated their current screening preference, 29 participants (52.7%) chose opportunistic screening.

Comparison before and after intervention

**Figure 1:**
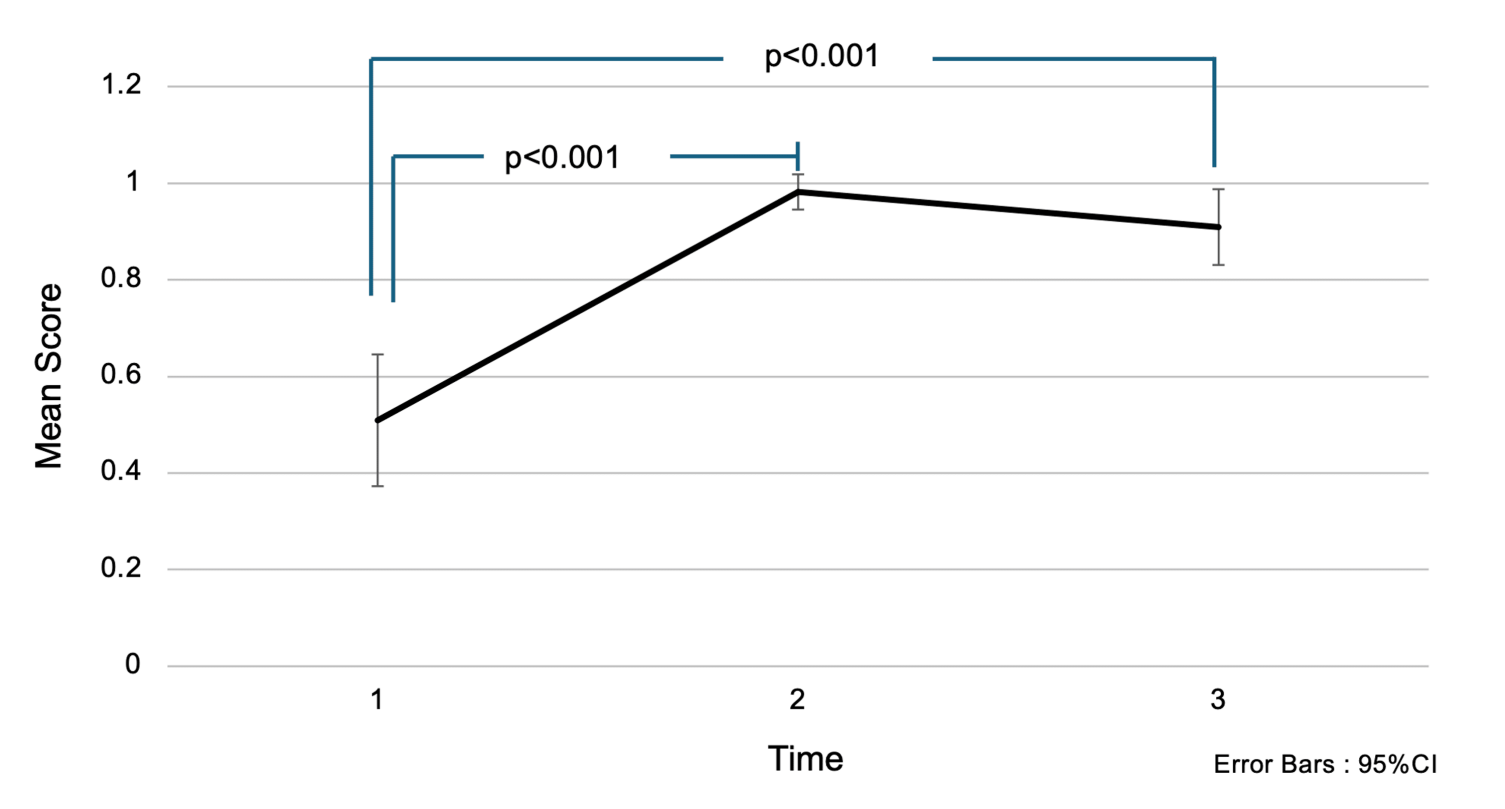
Changes in accuracy rates for the question: “Which of the following influences the division of 60-70% of breast cancer cells?” Choices: (1) Estrogen, (2) Progesterone, (3) Follicle-Stimulating Hormone. Analysis Method: One-way repeated measures ANOVA; multiple comparisons were conducted using the Bonferroni method. The numbers on the x-axis represent: 1 = Before the intervention, 2 = Immediately after the intervention, 3 = One and a half months after the intervention. Interpretation: Higher scores indicate greater knowledge. Initially, participants had low scores, reflecting limited knowledge. After the awareness education session, scores increased, indicating improved understanding.

Regarding the question “Which of the following affects the division of 60-70% of breast cancer cells?”, the mean score (± SD) was 0.51 (±0.51) before the intervention, 0.98 (±0.14) immediately after the intervention, and 0.91 (±0.29) one and a half months later (Figure 1). Multiple comparison tests revealed a significant difference in mean scores between before the intervention and immediately after the intervention (p<0.001). Before the lecture, only about half of the participants were aware of the luminal subtype of breast cancer, which grows in response to estrogen. After receiving the awareness education, their scores significantly increased, approaching a perfect score.

**Figure 2:**
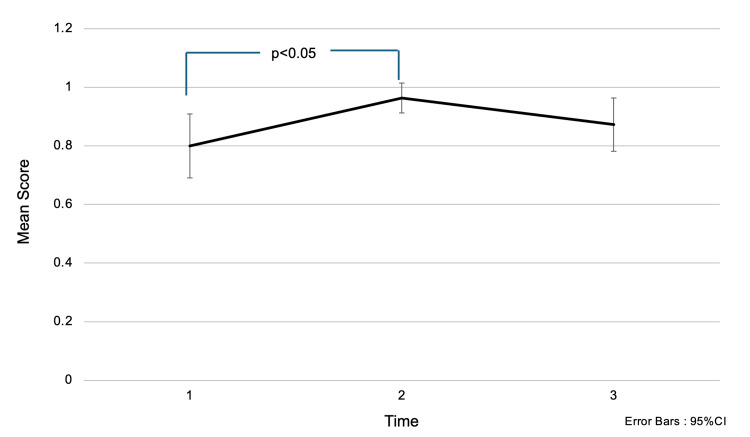
Changes in accuracy rates for the question: “What are the stages of breast cancer?” Choices: (1) Stage 0 to Stage II, (2) Stage 0 to Stage III, (3) Stage 0 to Stage IV. Analysis Method: One-way repeated measures ANOVA; multiple comparisons were conducted using the Bonferroni method. The numbers on the x-axis represent the following: 1 = Before the intervention, 2 = Immediately after the intervention, 3 = One month after the intervention. Interpretation: Higher scores indicate greater knowledge. Even before the intervention, participants had relatively high scores, suggesting a good level of prior knowledge. After the awareness education session, scores increased further, indicating improved understanding.

Regarding the question “Up to what stage is breast cancer classified?”, the mean score (± SD) was 0.80 (±0.40) before the intervention, 0.96 (±0.19) immediately after the intervention, and 0.87 (±0.34) one and a half months later (Figure 2). Multiple comparison tests revealed a significant difference in mean scores between before the intervention and one month after the intervention (p<0.05). Scores increased significantly immediately after the awareness education; however, participants already had a relatively high level of knowledge regarding cancer staging, with approximately 80% scoring correctly prior to the intervention.

**Figure 3:**
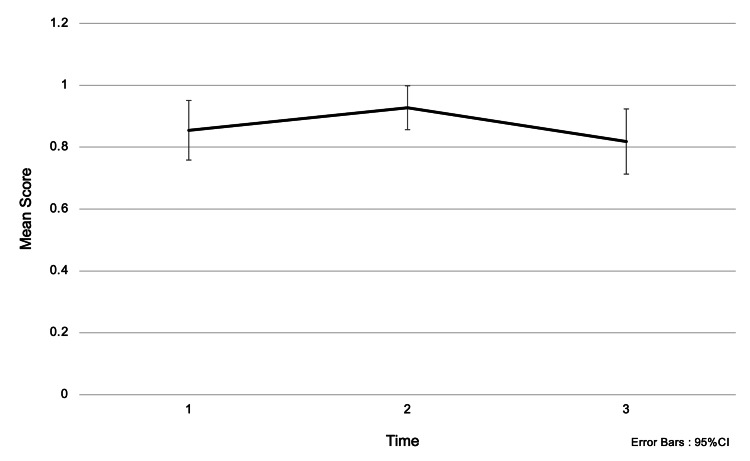
Changes in accuracy rates for the question: “Which of the following is considered early-stage cancer?” Choices: (1) Non-invasive cancer, (2) Invasive cancer. Analysis Method: One-way repeated measures ANOVA; multiple comparisons were conducted using the Bonferroni method. The numbers on the x-axis represent: 1 = Before the intervention, 2 = Immediately after the intervention, 3 = One and a half months after the intervention. Interpretation: The intervention did not result in a significant increase in knowledge scores. This suggests that participants already had a sufficient level of knowledge prior to the awareness education, thereby reducing the potential impact of the intervention.

Regarding the question “Which of the following is considered early-stage cancer?”, the mean score (± SD) was 0.85 (±0.36) before the intervention, 0.93 (±0.26) immediately after the intervention, and 0.82 (±0.39) one and a half months later. There were no significant differences among students before the intervention, on the day of the intervention, or one and a half months later (Figure 3). Understanding of early-stage cancer was consistent regardless of whether participants received the awareness education.

**Figure 4:**
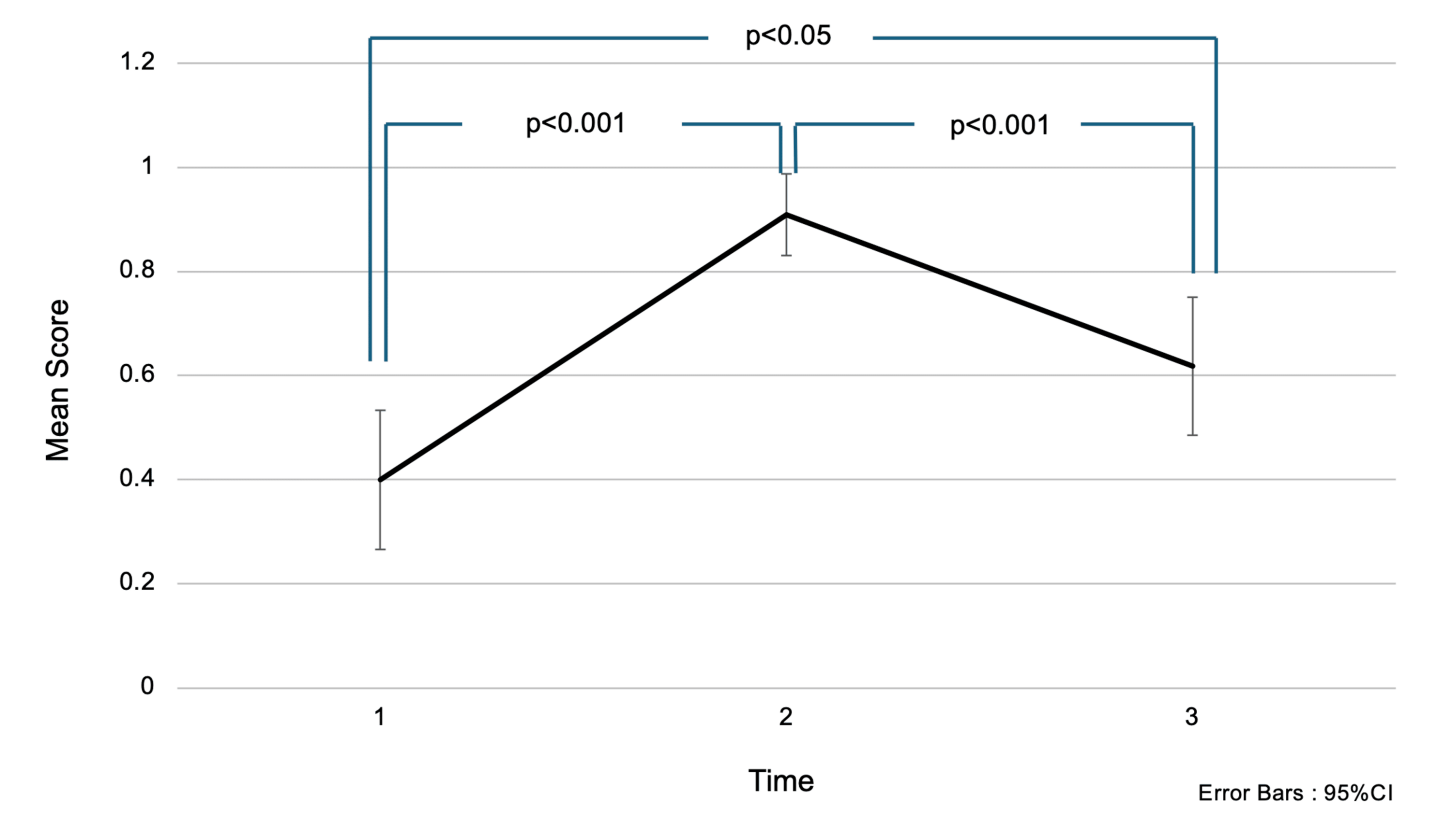
Changes in accuracy rates for the question: “At what age range does the first peak in the incidence of breast cancer occur in women?” Choices: (1) Late 20s, (2) Late 30s, (3) Late 40s, (4) Late 50s. Analysis Method: One-way repeated measures ANOVA; multiple comparisons were conducted using the Bonferroni method. The numbers on the x-axis represent: 1 = Before the intervention, 2 = Immediately after the intervention, 3 = One and a half months after the intervention. Interpretation: Higher scores indicate greater knowledge. Scores were low before the intervention but increased significantly immediately afterward. However, a decline was observed one and a half months later, suggesting that the learning effect was not retained over time.

Regarding the question “At what age range does the first peak in the incidence of breast cancer occur in women?”, the mean score (± SD) was 0.40 (±0.50) before the intervention, 0.91 (±0.29) immediately after the intervention, and 0.62 (±0.49) one and a half months later. Scores significantly decreased both immediately after the intervention and one and a half months later (p<0.001). Multiple comparison tests revealed a significant difference in mean scores between before the intervention and immediately after (p<0.001), as well as between immediately after and one and a half months later (p<0.001). Participants originally lacked knowledge about the age at which breast cancer becomes more prevalent, and the fact that incidence peaks in the 40s was easily forgotten. These results suggest that a single awareness education session was not sufficient for long-term retention of this knowledge (Figure 4).

**Figure 5:**
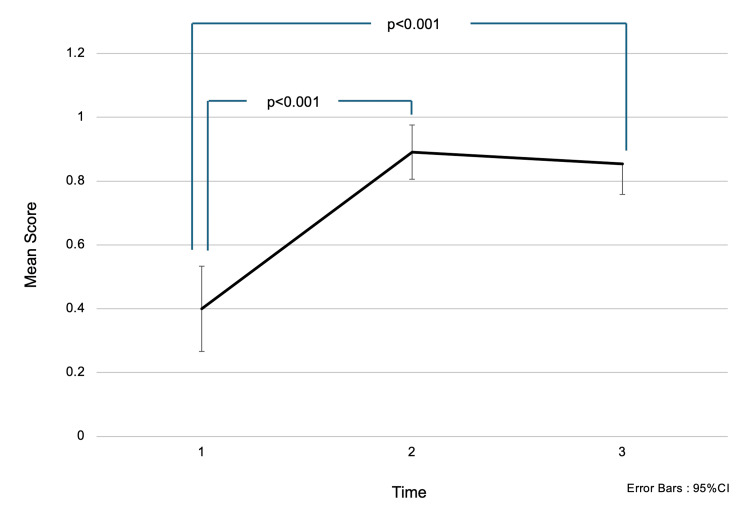
Changes in accuracy rates for the question: “Who is affected by hereditary breast and ovarian cancer syndrome among the following?” Choices: (1) Only women, (2) Only men, (3) Both sexes Analysis Method: One-way repeated measures ANOVA; multiple comparisons were performed using the Bonferroni method. The numbers on the x-axis represent: 1 = Before the intervention, 2 = Immediately after the intervention, 3 = One and a half months after the intervention. Interpretation: Higher scores indicate greater knowledge. Scores were low before the intervention but increased significantly immediately after the awareness education. This improvement was maintained over time, suggesting that the acquired knowledge was retained.

Regarding the question “Who is affected by Hereditary Breast and Ovarian Cancer Syndrome among the following?”, the mean score (± SD) was 0.39 (±0.49) before the intervention, 0.89 (±0.32) immediately after the intervention, and 0.85 (±0.36) one and a half months later. Multiple comparison tests revealed a significant difference in mean scores between before the intervention and immediately after (p<0.001), as well as between before and one and a half months after the intervention (p<0.001). Although scores slightly declined from immediately after the intervention to one and a half months later, the difference was not statistically significant (Figure 5). With regard to HBOC, few participants were aware of it prior to the intervention. After the educational session, the average score increased significantly. Although no significant decline was observed one month later, scores remained higher than baseline.

In Figures 1-2 and Figures 4-5, the one-way ANOVA revealed a significant difference in mean scores over time (p<0.001).

## Discussion

This study aimed to evaluate the effectiveness of an educational awareness program for the AYA generation, with the goal of promoting the long-term practice of breast awareness. While the program demonstrated a certain level of effectiveness, the results indicated that a single session was insufficient to sustain knowledge and awareness over the long term.

To examine the effectiveness of the awareness education program using slide-based materials, questionnaire surveys were conducted one month before the intervention, immediately after the intervention, and one month after the intervention. Scores for all questions were higher on the day of the intervention than before the intervention. A comparison with previous studies [18,19] showed no change in pre-intervention mean scores. In particular, for the question regarding “which genders can be affected by HBOC,” about 50% of participants lacked knowledge prior to the intervention. However, scores increased significantly immediately after the session. Previous studies have reported that education on genetic counseling related to HBOC improves knowledge and awareness, showing a trend similar to that observed in this study [22]. The results of the HBOC item in this study also suggest that even a single educational session can leave a lasting impression and convey essential information. Another previous study that examined awareness of HBOC by comparing individuals under and over the age of 39 found no significant differences in knowledge between the two age groups [23]. The reason for the low level of knowledge about HBOC before the educational intervention in this study is likely due to the participants’ young age, around 20 years old, when concerns about breast cancer tend to be low [24], resulting in a general lack of interest. It can be inferred that by the time individuals reach their late 30s, they may have acquired health-related information, including about HBOC, from sources such as the internet [23], and, as shown in this study, hearing about HBOC even once may be sufficient for them to at least recognize the term. Therefore, incorporating the term HBOC into educational materials such as pamphlets [25] and including HBOC content in awareness education programs may be a meaningful approach to promote breast awareness.

Although male breast cancer accounts for only 1% of all breast cancer cases, it has been linked to HBOC, particularly the BRCA1 and BRCA2 mutations. Since the average age at diagnosis is between 60 and 70 years [26], awareness education needs to emphasize the risk of developing breast cancer later in life, beyond the AYA generation. Furthermore, in response to the question “Who is affected by hereditary breast and ovarian cancer syndrome?”, the mean score doubled from before the intervention to one month after the intervention, showing a significant difference. Since HBOC also affects men [6], it is crucial to ensure understanding regardless of sex. These results confirm that this topic may be effectively retained after a single awareness education session. Therefore, it is essential to continue including this content in future awareness education programs.

For the item “At what age does the first peak of breast cancer incidence in women occur?”, approximately 50% of participants lacked this knowledge prior to the educational intervention. This finding is consistent with previous studies, which reported that about 70% of individuals were unaware of the increased risk in women over 40 years old [14]. Furthermore, in this study, the recognition of the peak incidence in women in their late 40s significantly declined from immediately after the intervention to one and a half months later, indicating that a single educational session may be insufficient for long-term understanding. In a previous study comparing those under 39 and those 40 and older, the latter group was significantly more likely to correctly identify that breast cancer screening starts at age 40 [23]. To promote breast awareness, four key components have been identified: becoming familiar with one’s own breasts by regularly observing, touching, and being aware of any changes; understanding which types of breast changes require attention; consulting a medical professional as early as possible when any changes are noticed; and undergoing regular breast cancer screening starting at age 40 [7]. However, for the question “At what age range does the first peak in the incidence of breast cancer occur in women?”, scores significantly decreased one and a half months after the intervention. This result suggests that the AYA generation may not fully understand the difference between organized screening and opportunistic screening, such as that conducted through comprehensive health check-ups or workplace medical examinations [7]. In Japan, the Ministry of Health, Labour and Welfare has established guidelines for cancer screening, under which municipalities provide scientifically based screening services. According to these guidelines, breast cancer screening targets women aged 40 and older, with a recommended interval of once every two years [27]. However, individuals in the AYA generation rarely have the opportunity to undergo screening while asymptomatic, and as a result, they are more likely to be diagnosed at a more advanced stage compared to other age groups [7]. Furthermore, Japan’s breast cancer screening rate among women aged 40 and older was only 44.6% in 2019, one of the lowest rates internationally [28]. Given these circumstances, including the message that “the first peak age of breast cancer incidence in women is in the late 40s” in awareness education programs may enhance their immediate effectiveness. However, as shown by the significant decline in knowledge one month after the intervention, maintaining this effect over the long term remains a challenge. Even before the intervention, the scores for this item were lower than those for other items. Therefore, participants may not be familiar with the meaning of population-based screening and opportunistic screening. Additionally, immediately after the awareness education session, only a few participants were able to correctly identify “luminal type.” These results indicate that the terminology used may be too difficult, highlighting the need to communicate information in a clearer and more understandable manner. Additionally, there may not have been sufficient time during the awareness education session to thoroughly explain why Japan adopts organized screening for women aged 40 years and older.

Among the participants in this study, only about half were aware before the intervention that 70-80% of breast cancers are hormone receptor-positive and that estrogen, a female hormone, binds to estrogen receptors to promote the proliferation of cancer cells [7]. Without an understanding of the hormonal changes associated with pregnancy and childbirth, it may be difficult to recognize “not having given birth” as a scientifically established risk factor. However, the findings of this study suggest that this concept can be understood once it is addressed in an educational session.

Furthermore, the lack of a significant difference in understanding early-stage cancer suggests that participants may already be familiar with the general concept of “early detection and early treatment,” which is commonly taught from a young age and not limited to breast cancer. As for breast cancer staging, the study showed that even brief exposure to the topic can lead to increased awareness and recognition.

This study demonstrated that although a small amount of knowledge gained after the awareness education led to improved scores, all scores declined one and a half months later. However, providing individuals with opportunities to participate in awareness education may help prevent disinterest in their own health [15] and potentially foster greater interest and engagement. Since the incidence of breast cancer begins to rise from the AYA generation, it is crucial to have at least a basic understanding of breast health to maintain breast awareness over time. To achieve this, it is necessary to provide multiple sessions of awareness education during the period before age 40, when no organized screening is available, and encourage individuals to seek medical attention when needed [7].

Breast awareness is recommended in Japan’s priority health education for cancer prevention [13] and is expected to contribute to an increase in the screening rate for population-based breast cancer screening among individuals aged 40 years and older. Therefore, repeated exposure to information may help instill breast awareness. Opportunities such as providing education to mothers attending 1.5-year-old and 3-year-old infant health check-ups need to be considered. Such efforts may contribute to earlier detection and treatment of breast cancer, which in turn could lead to a reduction in breast cancer mortality rates in Japan and a decrease in overall healthcare costs. Furthermore, as the Ministry of Health, Labour and Welfare promotes social security and workstyle reforms toward 2040, with a key policy goal of extending healthy life expectancy, strengthening outreach to populations with low health awareness has been emphasized. In this context, the findings of this study may have significant social impact [29].

In this study, only five items were used to assess participants’ knowledge before, immediately after, and one and a half months after the awareness education intervention, which limited the depth of insights that could be obtained. Additionally, the Cronbach’s alpha was 0.61, indicating that the reliability of the data was not high. Although the results may not be statistically robust, the findings suggest that without continued awareness education, knowledge tends to decline over time. Moreover, the small sample size and the fact that the study was conducted at a single institution highlight the need for larger-scale studies to enable standardization.

To enhance the outcomes that serve as evaluation measures for the effectiveness of the awareness education program, it is necessary to increase the number of questionnaire items and examine both their reliability and validity. Since the study was limited to nursing students and did not include a control group of general students, the findings cannot be directly generalized to the broader population, as many nursing students may already have a higher interest in breast cancer. There are several potential confounding factors, such as being healthcare students, differences in educational background, health literacy levels, and access to online information, that may have influenced the results. Future studies should include analyses that distinguish between healthcare students and general students. In this study, the focus was limited to five items: “breast cancer subtypes,” “HBOC,” “staging,” “peak age starting at 40,” and “invasive vs. non-invasive cancer.” Even when accounting for potential confounding factors, the result that the “peak age in the 40s” is easily forgotten suggests that this particular information may be especially difficult to retain.

This study was a cross-sectional investigation, and due to the limited number of participants and the small number of multidimensional questionnaire items, future research should consider longitudinal designs to explore causal relationships and identify effective methods for sustaining breast awareness. Therefore, this study should be regarded as a pilot study.

Despite these limitations, it is noteworthy that in Japan, no standardized awareness education programs for promoting breast awareness have yet been established. This study provides preliminary data that may serve as a foundational resource for developing such programs aimed at fostering breast awareness.

## Conclusions

The awareness education program aimed at promoting sustainable breast awareness demonstrated a certain level of effectiveness immediately after the intervention. However, the findings indicated that a single session was insufficient to maintain these effects over the long term. Future awareness initiatives should include information about the peak incidence of breast cancer in women in their 40s and be conducted repeatedly to improve knowledge retention and ensure long-term impact.

## Appendices

Appendix 1 

**Table 3:** Self-administered questionnaire conducted before the awareness education intervention. Correct answers are indicated in bold.

Question Items	Answer Choices
1	What year are you in? (1) 1st year (2) Other: ( ) year
2	How old are you? ( ) years old
3	Please indicate your sex (circle one). (1) Male (2) Female
4	Do you know someone close to you who has had breast cancer? (e.g., your mother, grandmother, sister, aunt, friend, or acquaintance) (1) Yes (2) No
5	Do you perform breast cancer self-examinations? (1) Yes (2) No
6	Which of the following affects the division of 60–70% of breast cancer cells? (1) Estrogen (2) Progesterone (3) Follicle-stimulating hormone
7	What are the stages of breast cancer? (1) Stage 0 to Stage II (2) Stage 0 to Stage III (3) Stage 0 to Stage IV
8	Which of the following is considered early-stage cancer? (1) Non-invasive cancer (2) Invasive cancer
9	At what age range does the first peak in the incidence of breast cancer occur in women? (1) Late 20s (2) Late 30s (3) Late 40s (4) Late 50s
10	Who is affected by Hereditary Breast and Ovarian Cancer Syndrome among the following? (1) Only women (2) Only men (3) Both sexes
11	Where do you obtain information about breast cancer? (1) Television/Radio (2) Internet (3) Newspapers/Magazines/Books (4) Information from government agencies (5) Pamphlets (6) School lessons (7) Participation in external awareness education (8) Other: ( )
12	Have you ever received breast cancer awareness education? (1) Yes (2) No
13	Would you like to attend breast cancer awareness education sessions in the future? (1) Yes (2) No

Appendix 2 

**Table 4:** Self-administered questionnaire administered immediately after the awareness education session. Correct answers are indicated in bold.

Question Items	Answer Choices
How did you find the difficulty level of today’s awareness education session?	(1) Difficult (2) Just right (3) Easy
How did you feel about today’s awareness education (breast cancer lecture content)?	(1) Useful (2) Neutral (3) It was all information I already knew (4) Other ( )
Regarding the slide materials:	(1) Easy to understand (2) Neutral (3) Difficult
Regarding the handouts for breast cancer awareness education:	(1) Easy to understand (2) Neutral (3) Difficult
Regarding the pace of the awareness education session:	(1) Too fast (2) Just right (3) Too slow (4) Other ( )
What do you think is the ideal duration for a breast cancer awareness education lecture for better understanding?	(1) 30 minutes (2) 60 minutes (3) 90 minutes (4) Other ( ) minutes
Which of the following affects the division of 60–70% of breast cancer cells?	(1) Estrogen (2) Progesterone (3) Follicle-stimulating hormone
What are the stages of breast cancer?	(1) Stage 0 to Stage II (2) Stage 0 to Stage III (3) Stage 0 to Stage IV
Which of the following is considered early-stage cancer?	(1) Non-invasive cancer (2) Invasive cancer
At what age range does the first peak in the incidence of breast cancer occur in women?	(1) Late 20s (2) Late 30s (3) Late 40s (4) Late 50s
Who is affected by Hereditary Breast and Ovarian Cancer Syndrome among the following?	(1) Only women (2) Only men (3) Both sexes
Which of the following is the most common subtype of breast cancer?	(1) Luminal (2) HER2-positive (3) Triple-negative
Would you like to participate in another awareness education session in the future?	(1) Yes (2) No
After today’s lecture, do you feel motivated to undergo breast cancer screening in the future?	(1) Yes (2) No
If you were to undergo breast cancer screening now, which type of screening would you choose?	(1) Population-based screening (2) Opportunistic screening
